# Colonic Strongyloides stercoralis With Multimodality Imaging, Endoscopic, and Histopathologic Correlation

**DOI:** 10.7759/cureus.111381

**Published:** 2026-06-23

**Authors:** Justin Hackett, Minal Jagtiani, Arvind Sangwaiya, Deepti Reddi

**Affiliations:** 1 Department of Laboratory Medicine and Pathology, University of Washington, Seattle, USA; 2 Department of Radiology, University of Washington, Seattle, USA; 3 Department of General Internal Medicine, University of Washington, Seattle, USA

**Keywords:** colon, eosinophilic microabscess, lamina propria, peripheral eosinophilia, strongyloides stercoralis

## Abstract

We present a case of symptomatic colonic strongyloidiasis diagnosed in an immunocompetent patient with chronic peripheral eosinophilia, with an absolute eosinophil count ranging from 0.71-1.47 × 10^3/µL since 2013. Initial evaluation for persistent eosinophilia, including random colon biopsies, demonstrated increased eosinophils and mast cells within the biopsy fragments. The patient was subsequently lost to follow-up. In December 2025, the patient presented to the emergency department with left lower quadrant abdominal pain and loose bowel movements. Computed tomography demonstrated diffuse bowel wall thickening. Follow-up colonoscopy performed approximately three months later revealed diverticulosis and flat polyps. Random biopsies obtained from the left colon demonstrated focal *Strongyloides stercoralis* larvae within eosinophilic abscesses in the lamina propria. The patient received treatment with ivermectin and albendazole, with subsequent resolution of symptoms. This case highlights *S. stercoralis *infection as a potential consideration in patients with persistent unexplained eosinophilia and illustrates the role of histopathologic evaluation in establishing the diagnosis.

## Introduction

*Strongyloides stercoralis* is a soil-transmitted nematode estimated to infect hundreds of millions of people worldwide, with the highest burden in tropical and subtropical regions, including parts of Latin America, sub-Saharan Africa, and Asia [1-3]. Outside endemic regions, infection remains clinically important among migrants, travelers, and other individuals with prior exposure in endemic areas [4,5].

Unlike many other helminths, *S. stercoralis* has a unique autoinfection cycle. Rhabditiform larvae may transform into infective filariform larvae within the host, allowing infection to persist for decades without repeated environmental exposure [5]. In immunocompetent patients, this often produces chronic, low-burden infection with intermittent gastrointestinal, pulmonary, or cutaneous symptoms. In immunocompromised patients, especially those receiving corticosteroids or those with human T-lymphotropic virus type 1 (HTLV-1) co-infection, the autoinfection cycle may accelerate and lead to hyperinfection syndrome or disseminated disease, both of which are associated with substantial morbidity and mortality [6].

The reproductive lifecycle of *S. stercoralis* most commonly involves the upper gastrointestinal tract, specifically the mucosa of the duodenum and proximal jejunum. Consequently, standard histopathological confirmation relies on upper endoscopic duodenal biopsies [7,8]. Colonic strongyloidiasis is an established clinical entity. Colonic involvement has classically been associated with more extensive infection or hyperinfection, particularly in immunocompromised patients, but it may also occur in chronic infection [9]. This case report highlights the critical role of radiologic imaging, endoscopy, and histologic identification of focal *S. stercoralis* larvae within eosinophilic microabscesses of the lamina propria in establishing the diagnosis and guiding appropriate therapy.

## Case presentation

A 61-year-old otherwise healthy man with a history of mild hyperlipidemia presented to the emergency department with a four-day history of progressive gastrointestinal distress, loose stools, and intermittent sharp left lower quadrant abdominal pain. He reported recently sharing a meal with family members, none of whom developed similar symptoms. The patient denied any history of immunosuppression, recent systemic corticosteroid use, or other immunomodulatory therapies.

Review of prior medical records demonstrated chronic eosinophilia dating back to 2013 with travel history to Ethiopia, with eosinophil counts ranging from 0.71-1.47 x 10^3/µL (reference range: 0-0.50 x 10^3/µL). Additional records revealed intermittent, unexplained episodes of gastrointestinal distress and loose stools from 2017. The prior colonoscopy had demonstrated isolated aphthous ulcerations with increased mucosal eosinophils and mast cells; however, no definitive parasitic organisms were identified at that time. Specific stool examination for ova and parasites (O&P) was ordered but was not completed, as the patient was subsequently lost to follow-up.

Upon physical evaluation, the patient was afebrile, with blood pressure, heart rate, and respiratory parameters resting well within normal baseline limits. The abdomen was soft but demonstrated mild localized tenderness to palpation over the left lower quadrant without rebound or guarding. Initial laboratory evaluation demonstrated a normal total white blood cell count with 18% eosinophils and absolute eosinophilic count of 1.39 x 10^3/µL. A comprehensive metabolic profile, including renal and liver function testing were within normal limits.

A contrast-enhanced computed tomography (CT) scan of the abdomen and pelvis was performed in the portal venous phase. The imaging revealed extensive colonic diverticulosis alongside mild, localized mural wall thickening tracking from the distal transverse colon through the descending colon and down to the superior rectum (Figure 1), and these findings were interpreted as acute colitis. The patient was managed conservatively with symptomatic care and discharged with strict instructions to seek close outpatient primary care follow-up.

**Figure 1:**
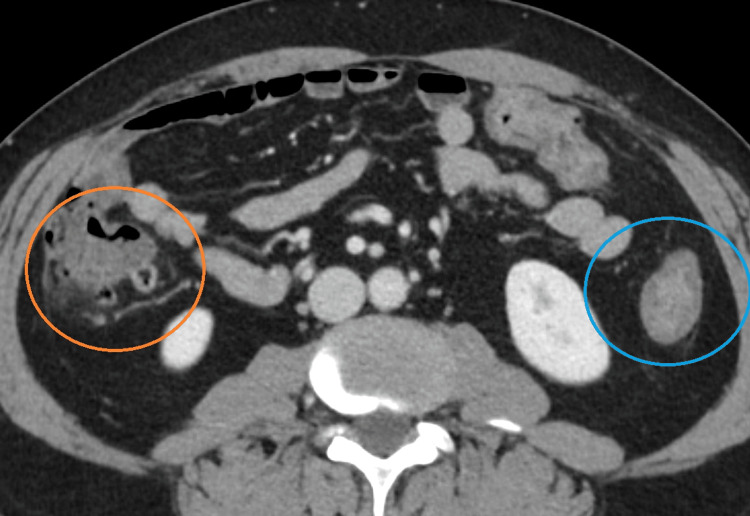
Axial contrast-enhanced computed tomography (CT) image of the abdomen and pelvis obtained in the portal venous phase. The image demonstrates colonic diverticulosis (orange circle), mild localized mural wall thickening (orange and turquoise circles), and subtle mucosal hyperenhancement spanning the distal transverse and descending colonic segments, suggestive of localized colitis.

Three months following his acute emergency presentation, the patient underwent an elective outpatient colonoscopy. Endoscopic visualization revealed an otherwise normal, healthy-looking colonic mucosa (Figure 2A) interspersed with multiple classic diverticula (Figure 2B) and two small, flat, benign sessile polyps measuring 4-6 mm. Random cold forceps tissue biopsies were taken from both the right and left colon for evaluation of microscopic colitis.

**Figure 2:**
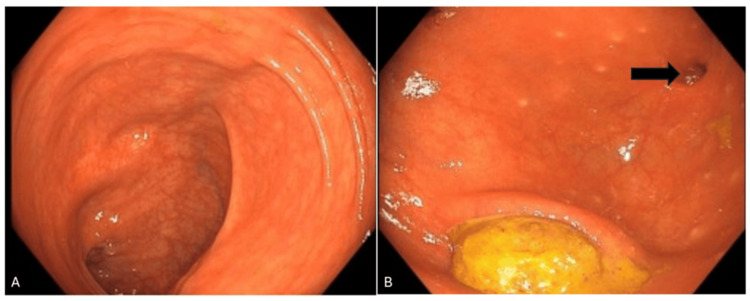
Diagnostic high-definition colonoscopy performed three months after initial presentation demonstrates normal-appearing colonic mucosa (A) with multiple uncomplicated diverticula (B, arrow) and no visible evidence of active inflammation.

Histopathologic examination of random colonic biopsies revealed a focal tissue fragment from the left colon containing prominent eosinophilic abscesses within the lamina propria. A rare but well-defined larval structure with basophilic stippling, consistent with *Strongyloides stercoralis*, was identified on hematoxylin and eosin (H&E), Giemsa, and periodic acid-Schiff (PAS) stained sections (Figure 3A-3C).

**Figure 3:**
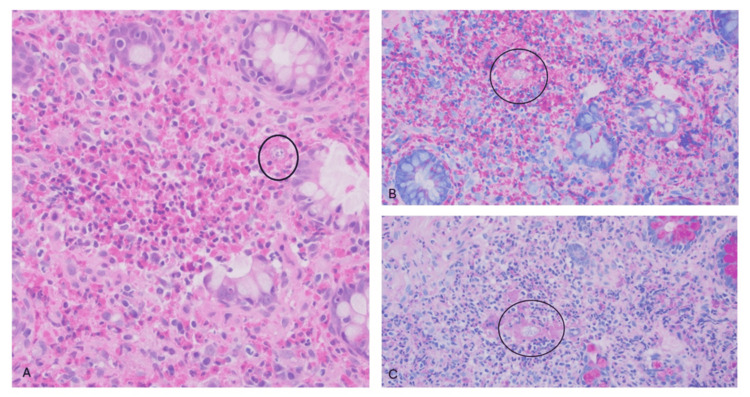
Histopathologic examination of the left colonic tissue biopsy fragment (200x magnification) revealing dense eosinophilic microabscesses within the lamina propria. Prominent rhabditiform larvae of Strongyloides stercoralis (black circles) are highlighted across H&E (A), Giemsa (B), and periodic acid-Schiff (PAS) (C) stained sections.

The patient was treated with a targeted dual antiparasitic regimen consisting of oral ivermectin, 12 mg as a single dose, and oral albendazole, 400 mg twice daily for one day. The treatment was well tolerated, with no reported adverse effects, and resolution of gastrointestinal symptoms at the one-month follow-up.

## Discussion

The clinical presentation of* S. stercoralis *in random lower-colon biopsies from an immunocompetent patient is an uncommon diagnostic finding. Typically, swallowed filariform larvae usually settle within the highly vascular, enzyme-rich environment of the duodenal and jejunal crypts, where parthenogenic females embed to lay eggs [10]. Colonic mucosa involvement is traditionally described in the literature as an advanced complication of severe hyperinfection [11]. In such states, massive larval expansion spills across all intestinal borders. However, our patient lacked any classic predisposing immunodeficiencies, suggesting that *S. stercoralis* can successfully set up secondary, long-term infection in the lower large intestine without necessarily causing systemic hyperinfection. Our case expands upon the observations of a prior cohort study by Minematsu et al., who noted that when symptomatic patients with known strongyloidiasis underwent full colonoscopy, colonic mucosal involvement was present in all cases, suggesting colonic invasion is a far more common, underappreciated aspect of the chronic, low-burden infection spectrum than previously thought [11].

The CT abnormalities identified in this case, characterized by non-specific mural thickening stretching from the transverse colon to the rectum, can potentially mislead clinicians. These features may overlap with more common acute diseases like idiopathic inflammatory bowel disease (IBD), ischemic colitis, or standard bacterial/diverticular colitis [12,13]. This diagnostic overlap can lead to devastating consequences; misdiagnosing parasitic colitis as an IBD flare often prompts immediate treatment with high-dose systemic corticosteroids, which can inadvertently trigger fatal hyperinfection syndrome [14]. An alternative explanation for the initial CT findings is an isolated flare of acute diverticulitis or concurrent bacterial colitis, which subsequently resolved prior to the outpatient colonoscopy. However, the patient’s chronic peripheral eosinophilia and subsequent histologic findings support *S. stercoralis *infection as a clinically relevant contributor.

Endoscopic evaluation performed three months after the acute presentation demonstrated normal-appearing colonic mucosa, with the exception of mild diverticular changes. This case underscores a key diagnostic pitfall; chronic helminthic infections may be present despite macroscopically normal mucosa. It also highlights the importance of obtaining random mucosal biopsies during endoscopic evaluation of unexplained lower abdominal pain or peripheral eosinophilia.

The identification of *S. stercoralis* larvae within localized eosinophilic microabscesses in a colonic biopsy is an exceptionally rare finding. In most cases, the organisms reside within mucosal crypts, where they may be detected on routine H&E examination [15]. When prominent eosinophilic infiltrates and Charcot-Leyden crystals are present in the lamina propria, deeper tissue levels and additional histochemical stains (e.g., PAS and Giemsa) may aid in identifying the parasite.

This case highlights the significant diagnostic delays that can occur in chronic strongyloidiasis. The patient had a documented long history of unexplained absolute eosinophilia, along with intermittent gastrointestinal symptoms. Routine stool examination for O&P is known to have limited sensitivity in low-burden infections, with a single specimen potentially missing up to 70% of cases due to intermittent and low-level larval shedding [16,17]. Although molecular and serologic methods, such as enzyme-linked immunosorbent assay (ELISA) for *Strongyloides *IgG, demonstrate higher sensitivity (approximately 75%-94%), their utility may be limited by cross-reactivity with other helminth infections [18]. In our patient, stool examination for O&P was not performed, as the patient was subsequently lost to follow-up.

While standard uncomplicated* S. stercoralis* infections are successfully treated using a short two-day course of oral ivermectin, our patient was successfully managed with a combination of ivermectin and albendazole [19,20]. This dual-therapy strategy led to complete clinical resolution of his gastrointestinal symptoms.

## Conclusions

Colonic strongyloidiasis is an underrecognized manifestation of chronic, low-burden disease that often leads to delayed diagnosis. This case highlights the potential diagnostic value of random colonic biopsies in establishing the diagnosis. Screening high-risk individuals prior to initiation of immunosuppressive therapy is essential to enable timely treatment and prevent progression to life-threatening hyperinfection syndrome.
